# Selective Reduction of Post-Selection CD8 Thymocyte Proliferation in IL-15Rα Deficient Mice

**DOI:** 10.1371/journal.pone.0033152

**Published:** 2012-03-20

**Authors:** Kai-Ping N. Chow, Jian-Tai Qiu, Jam-Mou Lee, Shuo-Lun Hsu, Shan-Che Yang, Ning-Ning Wu, Wei Huang, Tzong-Shoon Wu

**Affiliations:** 1 Department of Microbiology and Immunology, School of Medicine, Chang Gung University, Taoyuan, Taiwan, Republic of China; 2 Graduate Institute of Biomedical Sciences, School of Medicine, Chang Gung University, Taoyuan, Taiwan, Republic of China; 3 Department of Biomedical Sciences, School of Medicine, Chang Gung University, Taoyuan, Taiwan, Republic of China; 4 Department of Obstetrics and Gynecology, Chang Gung Memorial Hospital, Taoyuan, Taiwan, Republic of China; 5 Wellkent Biomedical Technology Inc., Taipei, Taiwan, Republic of China; University of Rochester, United States of America

## Abstract

Peripheral CD8^+^ T cells are defective in both IL-15 and IL-15Rα knock-out (KO) mice; however, whether IL-15/IL-15Rα deficiency has a similar effect on CD8 single-positive (SP) thymocytes remains unclear. In this study, we investigated whether the absence of IL-15 transpresentation in IL-15Rα KO mice results in a defect in thymic CD8 single positive (SP) TCR^hi^ thymocytes. Comparison of CD8SP TCR^hi^ thymocytes from IL-15Rα KO mice with their wild type (WT) counterparts by flow cytometry showed a significant reduction in the percentage of CD69^−^ CD8SP TCR^hi^ thymocytes, which represent thymic premigrants. In addition, analysis of *in vivo* 5-bromo-2-deoxyuridine (BrdU) incorporation demonstrated that premigrant expansion of CD8SP TCR^hi^ thymocytes was reduced in IL-15Rα KO mice. The presence of IL-15 transpresentation-dependent expansion in CD8SP TCR^hi^ thymocytes was assessed by culturing total thymocytes in IL-15Rα-Fc fusion protein-pre-bound plates that were pre-incubated with IL-15 to mimic IL-15 transpresentation *in vitro*. The results demonstrated that CD8SP thymocytes selectively outgrew other thymic subsets. The contribution of the newly divided CD8SP thymocytes to the peripheral CD8^+^ T cell pool was examined using double labeling with intrathymically injected FITC and intravenously injected BrdU. A marked decrease in FITC^+^ BrdU^+^ CD8^+^ T cells was observed in the IL-15Rα KO lymph nodes. Through these experiments, we identified an IL-15 transpresentation-dependent proliferation process selective for the mature CD8SP premigrant subpopulation. Importantly, this process may contribute to the maintenance of the normal peripheral CD8^+^ T cell pool.

## Introduction

The intrathymic development of T cells is a differentiation process in which bone marrow derived progenitors progress through an immature CD4^−^CD8^−^ double-negative (DN) TCR^neg^ stage, a CD4^+^CD8^+^ double-positive (DP) TCR^lo^ stage, and a mature CD4^+^ single-positive (SP) TCR^hi^ or CD8SP TCR^hi^ T cell stage [1]. In addition, a transitional CD8SP TCR^neg/lo^ stage exists prior to the DP stage [2]. Accompanying this differentiation process is a stage-wise cell expansion process. The earliest cell expansion takes place in DN thymocytes prior to TCR gene rearrangement [3]. Cell expansion also commences upon β-selection and continues through the proliferation-dependent DN to DP transition [4], [5]. Intriguingly, mature SP TCR^hi^ thymocytes that survive positive and negative selection undergo cell expansion several hours prior to their egress to the periphery. This premigration expansion takes place after the thymocyte selection processes and is therefore referred to as post-selection proliferation [1], [6], [7]. *In vivo* BrdU incorporation studies have demonstrated that post-selection proliferation accounts for approximately 4×10^5^ SP TCR^hi^ cells in adult mice [1], [8]; the majority of these cells are CD8SP thymocytes [1], [9]. Half of these cycling cells (2×10^5^) terminate their DNA synthesis upon egress to the periphery and account for 10% of the 2×10^6^ daily T cell output [1], [10], [11], demonstrating the significant contribution of post-selection proliferation to the peripheral CD8^+^ T cell pool.

IL-15 is an inflammatory cytokine identified on the basis of its ability to support T cell growth [12]. The receptor for IL-15 consists of α, β, and γ_c_ chains, with the β-chain and γ_c_ chain being shared by IL-2R and the receptors for IL-2, IL-4, IL-7, IL-9, and IL-21, respectively [13], [14]. It has recently been established that IL-15 must be autonomously transpresented by its proprietary IL-15Rα chain to the IL-15Rβγ_c_-bearing responding cells to exert its functional activities; this phenomenon is referred to as IL-15 transpresentation [15]–[18]. Previous studies showed that mice ablated of IL-15Rα bear similar absolute numbers of splenic white blood cells but 30% fewer lymph node (LN) cells compared with WT mice [19], [20]. Further analysis indicated that the percentage of CD8^+^ T cells was reduced by 50% in spleens, LNs, and blood in IL-15Rα KO mice. There are approximately 7×10^6^ CD8^+^ T cells in IL-15Rα KO spleen versus 14.7×10^6^ in WT mice counterparts. The average number of CD8^+^ T cells per LN in IL-15Rα KO and WT mice was 1.8×10^5^ and 4.8×10^5^, respectively [20]. Subsequent bone marrow chimerism studies involving reciprocal adoptive transfer of T cell-depleted bone marrow cells from wild type (WT) or IL-15Rα KO mice to irradiated recipients demonstrated an absolute requirement for IL-15 transpresentation for the maintenance of peripheral CD8^+^ T cells [21]. Despite these findings, whether IL-15 transpresentation is essential for thymic CD8^+^ T cell development and thus regulates the peripheral CD8^+^ T cell pool remains largely unknown. In previous studies, IL-15 was shown to stimulate the proliferation of CD8SP thymocytes that were isolated from mice deficient in suppressor of cytokine signaling 1 (SOCS-1), a negative regulator of cytokine signaling [22]–[27]. However, the *in vitro* proliferation of WT CD8^+^ SP thymocytes has been reported to require IL-15 and another γ_c_-dependent cytokine IL-21 [22], [26], [28]. The presence of IL-21 significantly enhances IL-15-mediated STAT5 phosphorylation, which may contribute to its synergistic effect on CD8^+^ T cell expansion [28].

To determine whether IL-15 supports CD8SP thymocyte expansion through transpresentation, which also contributes to the peripheral T cell pool, we directly compared the number of cells undergoing blastogenesis and the incorporation of 5-bromo-2-deoxyuridine (BrdU) by mature SP thymocytes *in vivo* in WT and IL-15Rα KO mice using flow cytometry. The requirement for IL-15 transpresentation for premigration expansion was also examined in *in vitro* cultures supplemented with pre-bound IL-15/IL-15Rα-Fc fusion protein. The contribution of premigration expansion to the peripheral T cell pool was subsequently examined by comparing the frequency of BrdU^+^ recent thymic emigrants (RTEs) identified by intrathymic fluorescein isothiocyanate (FITC) injection in WT and IL-15Rα KO mice.

## Materials and Methods

### Ethics statement

Animal ethics approval for the immunization studies in mice was obtained from the Institutional Animal Care and Use Committee (IACUC) at the Chang Gung University. All animal studies were performed in compliance with the guidelines of the Institutional Animal Care and Use Committee (IACUC) under approval from the IACUC at the Chang Gung University (Permit Number CGU 10-042).

### Mice and cell lines

IL-15Rα KO mice were previously generated as described [20] and backcrossed to C57BL/6 (B6) mice for 16 to 20 generations. Mice used in this study were bred under specific pathogen-free (SPF) conditions at the animal facility of Chang-Gung University. The experimental protocols conducted were approved by the Animal Ethics Committee, Chang Gung University. All mice were used at 6–8 weeks of age. The mouse lymphoma cell line EG7-IL-21H, which constitutively expresses IL-21 [29], was a gift provided by Dr. C.R. Shen of Chang Gung University, Taiwan.

### Medium and cytokines

RPMI-1640 medium (Invitrogen Life Technologies, Carlsbad, CA, USA) was supplemented with 10% heat-inactivated fetal calf serum (Biological Industries, Israel), 2 mM L-glutamine, 20 mM HEPES-NaOH (pH 7.2, Merck, NJ, USA), 2000 U/L penicillin-streptomycin (Invitrogen Life Technologies), and 50 µM β–mercaptoethanol (Merck). Recombinant mouse IL-15 was purchased from PeproTech, Inc. (PeproTech, Rocky Hill, NJ, USA). IL-21 was obtained from the supernatant collected 4 hours after culturing the IL-21-expressing cell line EG7-IL-21H [29]. The concentration of IL-21 was 6 ng/ml, as determined by a DuoSet ELISA kit (data not shown) (R&D Systems, Inc., Minneapolis, MN, USA).

### Flow cytometry

All cells were stained and analyzed using either a FACSCalibur or FACSCanto II flow cytometer (BD Biosciences, San Jose, CA, USA). For FACSCalibur analysis, cells were stained with anti- CD8α-FITC (clone 53-6.7), anti-CD4-biotin (clone GK1.5), and anti-TCRβ-PE (clone H57) in Mg^2+^- and Ca^2+^-free Dulbecco's phosphate buffer saline (PBS) containing 2% heat-inactivated FCS and 0.1% NaN_3_ (Life Technologies, Rockville, MD). The cells stained with biotin-conjugated Abs were washed and subsequently stained by allophycocyanin (APC)-conjugated streptavidin. The data acquired were then analyzed using Flow Jo software (Tree Star, Ashland, OR). For FACSCanto II analysis, cells were stained with anti-CD4-PE, anti-CD8-eFluor® 450, anti-TCRβ-FITC, anti-CD69-PE-Cy7 (clone H1.2F3) and anti-BrdU-APC. The cells stained with biotin-conjugated Abs were washed and stained with APC-conjugated streptavidin. All antibodies were purchased from eBioscience (San Diego, CA, USA).

### Isolation of SP thymocytes

Thymocytes were prepared as single-cell suspensions, and CD4^+^ thymocytes, including DP and CD4SP cells, were positively selected using CD4 (L3T4) MicroBeads (130-049-201, Miltenyi Biotec, Auburn, CA, USA) following the manufacturer's instructions. The flow-through was collected, and CD8SP thymocytes were positively selected using a CD8α^+^ T Cell Isolation Kit (130-090-859, Miltenyi Biotec). Isolated CD8SP thymocytes were routinely ≥90% pure.

### BrdU assay

To examine differences in cell division, WT and IL-15Rα KO mice were injected intraperitoneally (i.p.) with 100 µL of 10 µg/mL of BrdU (BD Biosciences) and sacrificed 1 hr later. Total thymocytes were collected and prepared as a single-cell suspension. For some experiments, CD8SP thymocytes were separated from CD4SP and DP thymocytes as described above. Cells were subsequently subjected to surface staining and permeabilized for intracellular staining of BrdU using a BrdU labeling kit (BD Biosciences).

### *In vitro* T cell proliferation assay

Total thymocytes were prepared as a single-cell suspension and labeled with 5 µM CFSE (Vybrant® CFDA SE Cell Tracer Kit, V-12883, Invitrogen Life Technologies, Molecular Probes). Cells (1×10^5^/mL) were then cultured in 96-well U-bottom plates containing 100 µL of medium supplemented with either 20 ng/ml murine IL-15 (PeproTech) or 6 ng/mL IL-21 or with both IL-15 and IL-21. For IL-15 transpresentation, wells were pre-coated with 3 µg/mL recombinant murine (rm) IL-15Rα-Fc fusion protein (R&D Systems) for 1 hr at 37°C. Plates were then blocked with PBS containing 5% serum for an equivalent length of time. IL-15 was added, and the plates were incubated for 1 hr at 37°C. Plates were then washed three times with PBS containing 5% serum. Next, total thymocytes were seeded in medium containing IL-21. Three days after seeding, the cultures were supplemented with an additional 100 µL of medium containing identical amounts of the same cytokine. Two days later, the cells were harvested and subjected to immunostaining and flow cytometric analysis.

### Detection of cycling RTEs by intrathymic FITC injection

Mice were injected twice with BrdU i.p.at a 2-hr interval to improve the labelling efficiency [1], [30]. Four hours after the second BrdU injection, mice were injected intrathymically with FITC as previously described [31]. Briefly, mice were anesthetized via an i.p. injection of 350 µL containing 0.75 mg ketamine hydrochloride and 350 µg xylazine (Sigma-Aldrich). The thoracic cavity was opened, and each thymic lobe was injected with 10 µL of 350 µg/mL FITC in 1× PBS. The chest was closed with a surgical staple, and the mice were warmed until fully recovered from anaesthesia. Mice were sacrificed 16 hrs after the BrdU injection by CO_2_ asphyxiation, and thymuses and lymph nodes (LNs) were removed. LN cells were subsequently stained for the expression of CD4 and CD8 and subjected to a BrdU assay. Flow cytometric analysis demonstrated that 60% of thymic cells were routinely labeled with FITC in both WT and IL-15Rα KO mice.

### Statistical analysis

Data are expressed as means ± SD and were compared among groups by single-classification ANOVA. A value of *p*<0.05 was considered to be statistically significant.

## Results

### Thymocytes develop normally in the absence of IL-15 transpresentation

**Figure 1:**
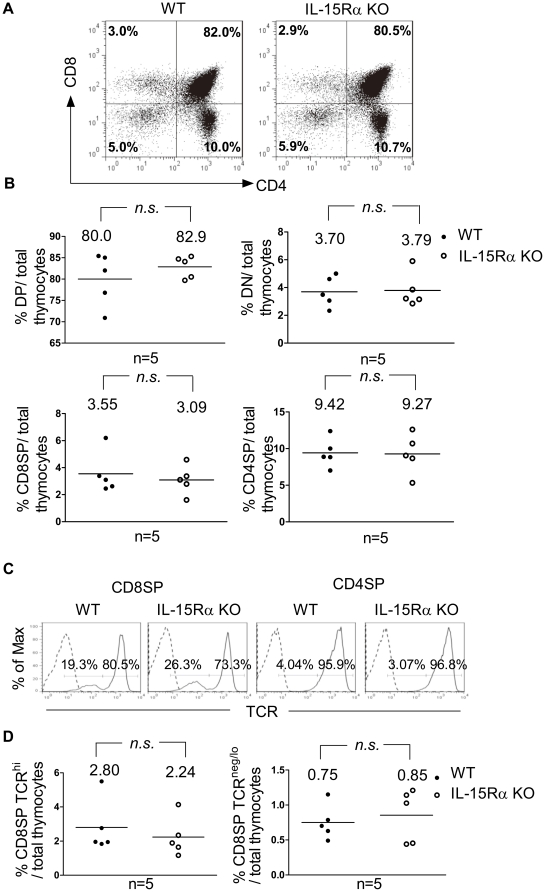
Characterization of thymocyte subpopulations in WT and IL-15Rα KO mice. Total thymocytes were isolated from WT and IL-15Rα KO mice (n = 5 per group) and subjected to flow cytometric analysis of the expression of CD4, CD8, and TCRβ. (A) Thymic subsets of WT and IL-15Rα KO mice were shown for their expression of CD4 and CD8. (B) Percentages of DN, DP, CD4SP, and CD8SP thymocytes among total thymocytes of IL-15Rα KO (○) mice and wild-type littermates (•) were compared. The horizontal line represents the mean. (C) CD4SP and CD8SP thymocytes were further gated to compare their TCR expression. CD8SP thymocytes consisted of TCR^neg/lo^ and TCR^hi^ cells, whereas the majority of CD4SP cells expressed high levels of TCR. (D) The percentage of CD8SP TCR^hi^ cells (left panel) and CD8SP TCR^neg/lo^ cells (right panel) among total thymocytes was compared between WT and IL-15Rα KO mice. Data were analyzed by single-classification ANOVA. n.s., not significant.

To determine whether there is a defect in intrathymic T cell development in the absence of IL-15 transpresentation, total thymocytes were isolated from WT and IL-15Rα KO mice and immunostained for CD4, CD8, and TCR to compare the frequencies of the DN, DP, and SP subpopulations in these mice. As shown in Fig. 1A and 1B, the compositions of the intrathymic DN, DP, CD4SP and CD8SP subpopulations were comparable between the two mouse strains. Furthermore, the majority of CD4SP thymocytes (≥95%) were TCR^hi^ mature cells (Fig. 1C, right two panels). In contrast, the CD8SP subpopulation was composed of approximately 80% mature TCR^hi^ cells and 20% immature cells with low or negative TCR expression (Fig. 1C, left two panels) [2]. Further comparison of CD8SP cells in these two groups of mice showed that the total thymocytes population contained similar percentages of immature CD8SP TCR^neg/lo^ and mature CD8SP TCR^hi^ cells in both mouse strains (Fig. 1D). Together with our previous finding that the frequency of cells in the four DN stages defined by the expression of CD44 and CD25 did not differ between WT and IL-15Rα KO mice [20], these results demonstrate that T cells develop in an IL-15 transpresentation-independent manner from DN progenitors to mature SP thymocytes.

### IL-15Rα KO thymus was selectively impaired in CD8SP premigrants

**Figure 2:**
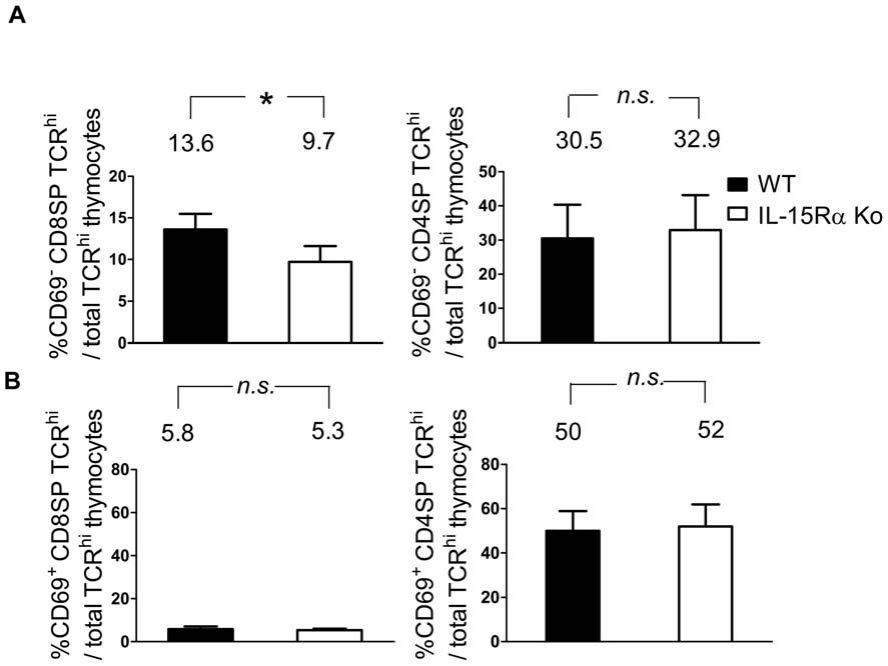
Examination of CD69-negative SP thymocytes in WT and IL-15Rα KO mice. Thymocytes of WT and IL-15Rα KO mice (n = 6) were immunostained for CD4, CD8, TCR, and CD69. The percentages of CD69-negative (A) and CD69-positive (B) CD8SP TCR^hi^ (left panels) and CD4SP TCR^hi^ thymocytes (right panels) among total TCR^hi^ cells were compared. By calculation, the cell numbers of CD69^−^ CD8SP TCR^hi^ thymocytes in WT and IL-15Rα KO mice were about 1.6×10^6^±3.8×10^5^ and 9.0×10^5^±2.8×10^5^, respectively. The cell numbers of CD69^−^ CD4SP TCR^hi^ thymocytes in WT and IL-15Rα KO mice were about 3.6×10^6^±1.3×10^5^ and 3.2×10^6^±1.4×10^5^, respectively. Data are presented as means ± SD and were analyzed by single-classification ANOVA. *p<0.05; n.s., not significant.

CD69 was known for its upregulation on newly activated peripheral T cells. However, DP thymocytes that were undergoing or have just undergone positive selection were also found to express CD69 [32], [33]. It was reported that CD69 mediated the suppression of cell surface sphigosine-1-phosphate receptor 1 (S1P1), a receptor essential for mature thymocyte emigration. CD69 downregulation was therefore a regulatory step for thymocyte development prior to their egress [33]–[35]. To determine whether the reduction of peripheral CD8^+^ T cells in IL-15Rα KO mice [20] was a result of a compromised supply from the thymus, we compared the thymic premigrants defined by the downregulation of CD69 within SP TCR^hi^ thymocytes in WT and IL-15Rα KO mice. IL-15Rα KO mice displayed a significant reduction in CD69^−^ CD8SP TCR^hi^ thymocytes (Fig. 2A). However, the CD69^−^ CD4SP TCR^hi^ thymocytes were unaffected. The frequency of mature CD69^+^ CD4SP and CD69^+^ CD8SP thymocytes was comparable in WT and IL-15Rα KO mice (Fig. 2B). Therefore, the mature CD8SP premigrant subpopulation is selectively impaired in IL-15Rα KO mice.

### The selective defect in CD8SP thymocytes was most prominent in blast cells

**Figure 3:**
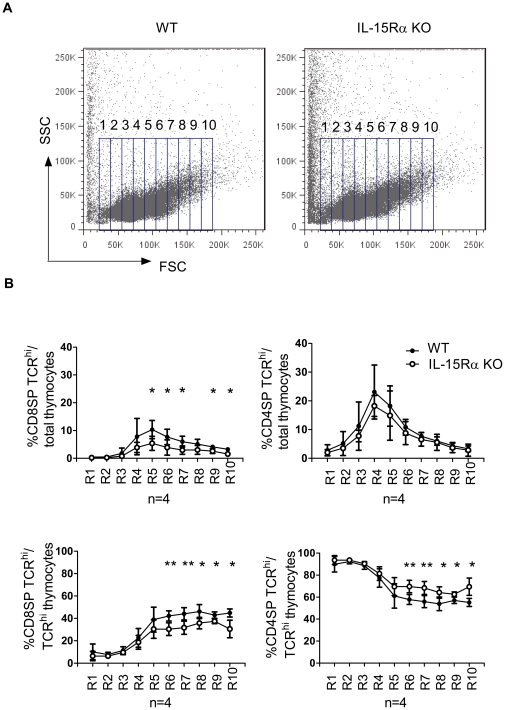
Comparison of large SP TCR^hi^ thymocytes between WT and IL-15Rα KO mice. Total thymocytes were isolated from WT and IL-15Rα KO mice (n = 4) and stained for CD4, CD8, and TCR. (A) Cells were gated into 10 sub-regions (R1 to R10) on the basis of FSC. (B) The frequency of CD8SP TCR^hi^ thymocytes in each specified regions is displayed as percentages of total thymocytes (upper panels) or percentages of total SP TCR^hi^ thymocytes (lower panels). Data are means ± SD values. **p<0.005 *p<0.05 Single-classification ANOVA.

It was believed that cycling SP thymocytes started egress approximately four hours after the initiation of DNA synthesis [1], [8]. We therefore investigated whether premigrant expansion is also an IL-15 transpresentation-dependent process. Given that cell expansion is accompanied by blastogenesis [6], [8], we first compared WT SP TCR^hi^ thymocytes with their IL-15Rα KO counterparts based on their cell size. Total thymocytes were first subdivided into ten subpopulations based on their forward scatter (FSC) by flow cytometry (Fig. 3A). The percentages of CD4SP TCR^hi^ or CD8SP TCR^hi^ cells among the total thymocyte population and among the TCR^hi^ thymocyte subpopulations were further analyzed. The results showed that the percentage of large CD8SP TCR^hi^ thymocytes in IL-15Rα KO mice (R5 to R10: 1.1×10^6^±3.8×10^5^, n = 4) was significantly reduced as compared to their WT counterparts (6.2×10^6^±5×10^5^ cells, n = 4) (Fig. 3B, left two panels). In contrast, the percentage of CD4SP cells among total thymocytes in each gated subpopulation remained comparable between WT (2.1 10^6^±1.9×10^6^ cells) and IL-15Rα KO (1.7×10^6^±1.5×10^5^) mice (Fig. 3B, upper right panel). However, when TCR^hi^ thymocytes were specifically analyzed, a significant increase of large CD4SP thymocytes was observed (Fig. 3B, lower right panel). As TCR^hi^ subpopulation is composed exclusively of CD4SP and CD8SP cells, the inverse change of the percentage respectively further indicated that IL-15Rα KO CD8SP TCR^hi^ thymocytes may be defective in post-selection cell proliferation. The cells larger than R-10 were excluded from analysis due to the extremely low percentages of SP thymcoytes (<2%) and significant variation between mice in this region.

### The reduction could be detected by in vivo BrdU incorporation

**Figure 4:**
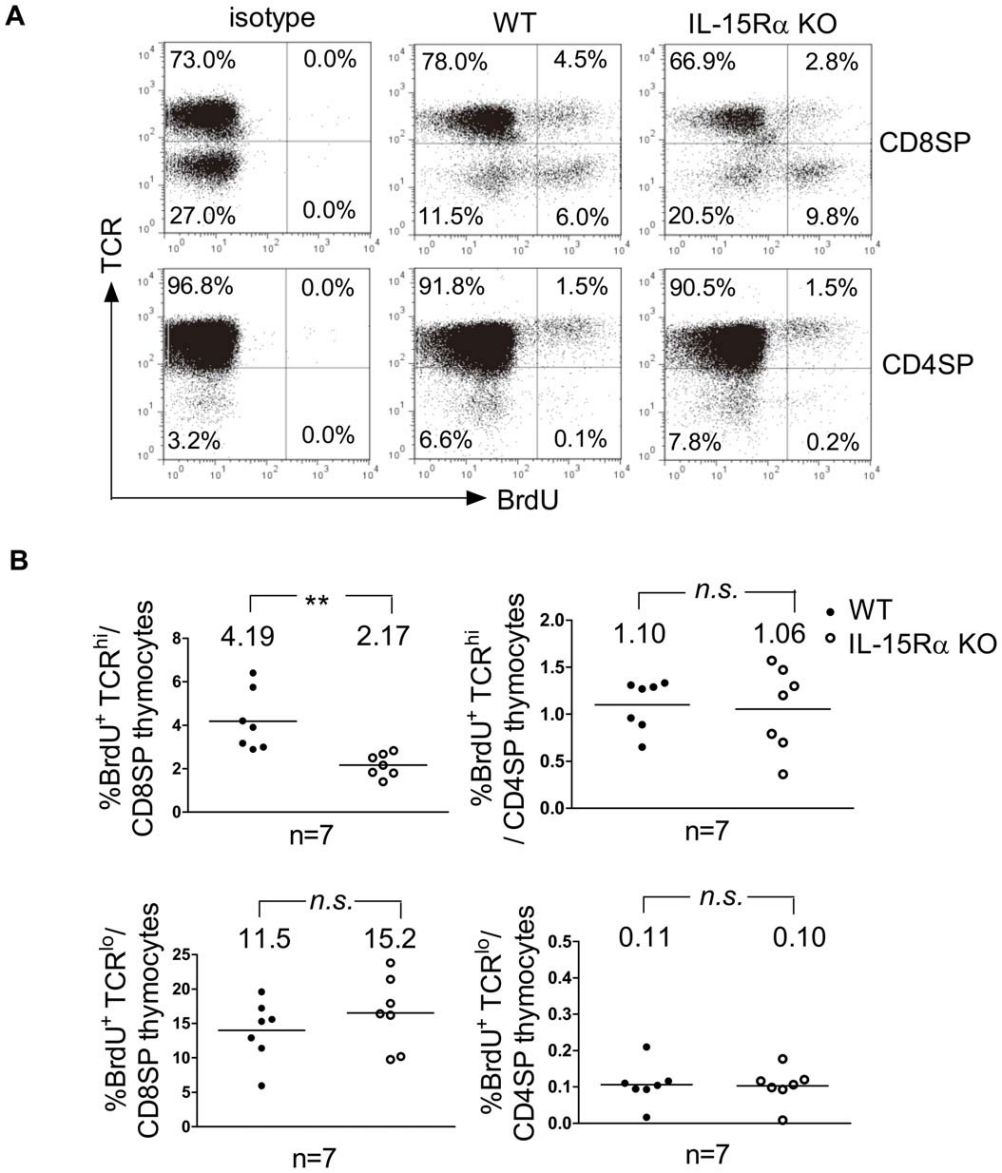
*In vivo* proliferation of CD8SP TCR^hi^ and CD4SP TCR^hi^ thymocytes analyzed by BrdU incorporation. WT and IL-15Rα KO mice (n = 7) were injected i.p. with BrdU. Total thymocytes were isolated 1 hr later, and the degrees of BrdU incorporation by CD4SP TCR^hi^ and CD8SP TCR^hi^ cells was determined. (A) Upper panel; The BrdU incorporation of IL-15Rα KO CD8SP TCR^hi^ cells (3.2×10^4^±1.1×10^3^ cells) was compared with that of WT CD8SP TCR^hi^ cells (9.4×10^4^±3.1×10^3^ cells). Lower panel; The percentages of WT (1.6×10^5^±1.0×10^5^ cells) and IL-15Rα KO CD4SP TCR^hi^ (1.5×10^5^±0.7×10^5^ cells) thymocytes that incorporate BrdU. (B) Values of each experimental group are averaged and the means are indicated by horizontal lines. Data are presented as means ± SD. **p<0.005; n.s., not significant. Single-classification ANOVA.

To further validate the defect in post-selection cell proliferation observed in IL-15Rα KO CD8SP TCR^hi^ thymocytes, WT and IL-15Rα KO mice were i.p. injected with BrdU. Total thymocytes were collected one hour later, and the frequency of BrdU^+^ cells within the CD4SP TCR^hi^ and CD8SP TCR^hi^ thymocyte subpopulations was determined by flow cytometry. Whereas the percentages of CD4SP TCR^hi^ thymocytes incorporating BrdU were comparable in WT and IL-15Rα KO mice (Fig. 4A, lower panel and 4B, upper right panel), IL-15Rα KO CD8SP TCR^hi^ thymocytes proliferated to a significantly lesser extent than their WT counterparts (Fig. 4A upper panel and 4B, upper left panel). Immature SP thymocytes proliferated equally well in WT and IL-15Rα KO mice (Fig. 4B lower two panels). These results imply that the intrathymic expansion of mature CD8SP thymocytes may be dependent in part on IL-15 transpresentation. In contrast, the ability of CD4SP thymocytes to proliferate is IL-15-independent.

### CD8SP thymocyte expansion is IL-15 transpresentation-dependent

**Figure 5:**
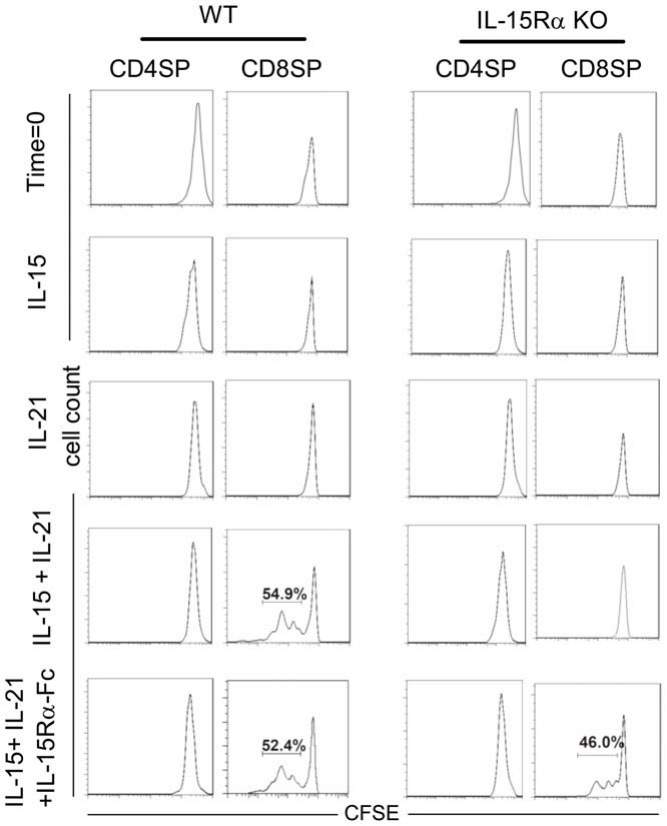
*In vitro* stimulation of CD8SP thymocyte proliferation with plate-bound IL-15-IL-15Rα-Fc proteins. Total thymocytes were labeled with CFSE and then cultured in medium supplemented with IL-15, IL-21, both IL-15 and IL-21, or both IL-15 and IL-21 with pre-coated rmIL-15Rα-Fc fusion protein. The cultures were supplemented with the same amounts of cytokine 3 days later. Cells were harvested 5 days after the initiation of the culture and stained for CD4 and CD8. The extent of cell proliferation in CD8SP thymocytes was analyzed by measuring CFSE dilution. The data are representative of three independent experiments.

The binding of IL-15 to its proprietary receptor leads to a conformational change in the cytokine. This conformational change in IL-15 enhances its functional activity when bound to the complex formed by the IL-15Rβ and γ_c_ chains [36]. Thus, presentation of IL-15 pre-associated with plate-bound IL-15Rα-Fc fusion protein has been used in *in vitro* studies to demonstrate IL-15 transpresentation-dependent proliferation of βγ_c_-bearing NK cells and peripheral CD8^+^ T cells [37]–[39]. However, neither IL-15 nor IL-21 alone but the combination of the two cytokines was able to support *in vitro* CD8^+^ T thymocyte growth [22], [26], [28]. Therefore, to investigate the dependence of CD8SP thymcoyte proliferation on IL-15 transpresentation, total thymocytes isolated from WT and IL-15Rα KO mice were labeled with CFSE and cultured in medium containing IL-15, IL-21, or IL-15 and IL-21 in the presence or absence of plate-bound IL-15Rα-Fc fusion protein. Five days later, cells were harvested and immunostained for CD4 and CD8, and the intensity of CFSE dilution in each cell subset were analysed by flow cytometry. As shown in Fig. 5, while WT CD8SP thymocytes did not exhibit significant proliferation in medium containing IL-15 or IL-21 alone, cells expanded in medium containing both cytokines. IL-15Rα KO CD8SP thymocytes exhibited proliferation only when IL-15 was transpresented by plate-bound IL-15Rα in the presence of IL-21. Neither WT nor IL-15Rα KO CD4SP thymocytes proliferated in either condition. These results provide direct evidence that the post-selection cell proliferation of mature CD8SP thymocytes but not CD4SP thymocytes was dependent on IL-15 transpresentation.

### Reduction in proliferating IL-15Rα KO CD8^+^ RTEs

**Figure 6:**
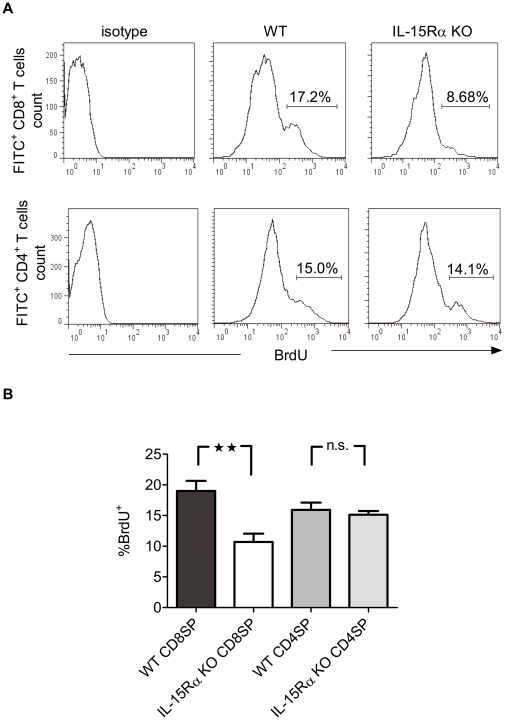
The dependence of CD8^+^ RTEs on IL-15 transpresentation. Mice were injected twice i.p.with BrdU at a 2-hr interval. Four hours after the second BrdU injection, mice were injected intrathymically with FITC or subjected to a sham operation (n = 6). (A) Cells were collected from the LNs 16 hours later and immunostained for the expression of CD8 (left panels) and CD4 (right panels). (B) The frequencies of BrdU^+^ FITC^+^ CD8^+^ or BrdU^+^ FITC^+^ CD4^+^ LN cells among FITC^+^ RTEs were compared. **p<0.005; n.s., not significant.

As cycling thymic premigrants stop DNA synthesis upon engaging in emigration process and subsequently become peripherally resident RTEs [1], post-selection cell proliferation is considered a significant contributor to the peripheral T cell pool. To determine whether RTEs that have recently undergone cell cycle were also selectively diminished in the periphery of IL-15Rα KO mice, we examined the frequency of cycling cells among RTEs from WT and IL-15Rα KO mice. Mice were injected twice i.p. with BrdU at a 2-hr interval. Four hours after the second BrdU injection, mice were injected intrathymically with FITC. Cells from axillary, inguinal, and mesenteric LNs were collected 16 hr later, immunostained for CD4 and CD8 and analyzed by flow cytometry. RTEs that had recently undergone cell cycle in the thymus were identified by incorporation of both FITC and BrdU (Fig. 6A). Whereas the percentages of FITC-gated BrdU^+^ CD4^+^ RTEs were similar in WT and IL-15Rα KO mice, FITC^+^ BrdU^+^ CD8^+^ RTEs were significantly reduced in IL-15Rα KO mice (Fig. 6B). These results suggest that the selectively reduced proliferation of IL-15Rα KO CD8SP thymocytes was also reflected in peripheral CD8^+^ T cells, potentially contributing to the diminished peripheral CD8^+^ T cell pool in IL-15Rα KO mice.

## Discussion

In this study, we have demonstrated for the first time a unique IL-15-dependent cell expansion at the mature CD8SP stage of thymocyte development. This phenomenon was observed through multiple experiments focusing on CD69 downregulation, cell size and BrdU incorporation of mature CD8SP thymocytes. Together with experiments utilizing *in vitro* culture conditions that mimic IL-15 transpresentation and experiments tracing the appearance of thymically FITC-labeled BrdU^+^ CD8^+^ T cells in the lymph nodes, these finding provide important physiological insights into the role of IL-15 in CD8^+^ T cell biology.

It has been established that IL-15 is indispensable for the maintenance of peripheral CD8^+^ T cells [19], [20], [40], which is achieved through the ability of IL-15 to support naïve peripheral CD8^+^ T cell survival rather than their homeostatic proliferation [20], [41]. Although this study demonstrated the IL-15-dependent proliferation of CD8SP thymocytes, previous studies showed that IL-15 alone was unable to support the growth of CD8SP thymocytes unless the negative regulator SOCS-1 was ablated [23], [25]. This study and others [23], [42] have demonstrated that stimulation with IL-15 and IL-21 induced the expansion of CD8SP thymocytes, suggesting that a synergistic effect upon STAT5 phosphorylation by IL-15 and IL-21 may override the negative effect of SOCS-1 and drive CD8SP thymocyte expansion [23], [43]. IL-21 alone acts through the induction of STAT1 and STAT3 in peripheral CD8^+^ T cells; however, IL-21 is not sufficient to induce CD8^+^ T cell expansion even in the absence of SOCS-1 [44]. The essential role of STAT5 phosphorylation in the IL-15-dependent proliferation of CD8SP thymocytes is in contrast to the lack of significant phosphorylation by IL-15 in CD4SP thymocytes in either the presence or absence of SOCS-1 [23], [27], underlining the restriction of this phenomenon to the CD8SP subset. In addition to inhibiting negative regulation or positively enhancing the STAT5 signals, a study of human CD8SP thymocytes cultured for 3 weeks suggests that the continuous addition of IL-15 may also drive CD8SP thymocytes into cell cycle [45]. However, proliferation of the culture cells was not specifically measured; the possibility that IL-15 plays a role in supporting cell survival cannot be excluded.

The extent of peripheral TCR diversity was significantly contributed by T cell progenitor expansion [4], [5] that was promoted by cytokine receptor signaling [46], [47]. Analysis of gene-ablated mice has shown that the expansion of the earliest progenitors CD44^+^CD25^−^ DN thymocytes is dependent on *c-kit* [48]. The blockade of T cell development at the following CD44^+^CD25^+^,CD44^−^CD25^+^, and CD44^−^CD25^−^ DN stages in γ_c_^−/−^ mice indicated that γ_c_ cytokine family members are also crucial for the development of DN thymic progenitors [12], [49], [50]. Mice deficient in IL-7 or IL-7Rα and double knock out of IL-7Rα and IL-2, or IL-7Rα and IL-4 demonstrated a similar blockade at the CD44^+^CD25^+^ DN stage, suggesting that IL-7, but not other γ_c_-dependent cytokines play an important role in the development of DN thymic progenitors [48], [51]. This notion was further supported by the normal T cell development in mice deficient in IL-2 [52], IL-2Rα [53], IL-4 [54], and IL-15Rα [20] and mice deficient in both IL-2 and IL-4 [55]. In addition, a body of evidence has been attributed to the additional diversity of TCR repertoire and the maintenance of the peripheral T cell pool to the expansion of mature thymocytes [1], [6], [56], [57]. IL-7 has been shown essential for the expansion of both CD4SP and CD8SP mature thymocytes [7], [58]. This report demonstrated that CD8SP thymocytes require an additional IL-15-dependent step to multiply in number before egress into circulation.

As for the cell types that transpresent IL-15 in the thymus, a previous study showed that mouse thymic epithelial cells (TECs) were able to drive mature thymocyte expansion [6]. Semiquantitative RT-PCR results also suggest that these cells constitutively express IL-15 [58], [59]. Moreover, TECs have recently been reported to transpresent IL-15 to support thymic cell development [60]. It is therefore possible that the unique expansion of mature CD8SP T cell premigrants is triggered through the IL-15 signal delivered by thymic stromal cells such as TECs.

Failure to maintain adequate peripheral TCR diversity and pool size is often evident in the elderly due to ageing-related thymic involution that is further exacerbated by diseases arising from chemotherapy, radiotherapy, or viral infection [6]. The subsequent homeostatic proliferation of the residual TCR repertoire-restricted peripheral T cells [61], [62] leads to the establishment of large CD8^+^ T cell clones [63] that results in a bias towards autoreactivity and increased incidence of autoimmunity [56], [64]–[68] and a decline in T-cell function [69]. Use of IL-7 has provided a future thymic rejuvenation strategy [67], [70], [71] for the treatment of thymic atrophy-associated immune problems and lymphopenia-related diseases. This study indicates that IL-15 could be an additional candidate to rejuvenate the thymus and maintain TCR repertoire that warrants further investigation via the use of aged WT and IL-15Rα KO mice.
